# Unclassified COL6A1 Variant in a Child With Congenital Hypotonia: A Case Report

**DOI:** 10.1002/ccr3.73765

**Published:** 2026-10-17

**Authors:** Khadeeja Nofal, Hamza M. A. Ghaith, Haneen Abdullatif Barghouthi, Mohammad Shahrori, Batool Darraj, Anwar Abu Hetta

**Affiliations:** ^1^ College of Medicine and Health Sciences Palestine Polytechnic University Hebron Palestine; ^2^ Department of Pediatrics Alia Governmental Hospital Hebron Palestine

**Keywords:** case report, COL6A1, congenital hypotonia, Ullrich congenital muscular dystrophy, variant of uncertain significance, whole exome sequencing

## Abstract

We report a 3‐year‐old male with congenital hypotonia, proximal contractures, and distal hyperlaxity. Exome sequencing identified a novel homozygous *COL6A1* variant (c.2821C>T), classified as a variant of uncertain significance. The distinct phenotype strongly supports Ullrich congenital muscular dystrophy, expanding the gene's mutational spectrum in consanguineous populations.

## 1 Introduction

Collagen type VI is a ubiquitous microfibrillar collagen predominantly found in the extracellular matrices of skeletal muscles and connective tissues. It plays a critical role in maintaining the structural integrity and elasticity of blood vessels, skin, and joints, interacting with integrins and regulating various growth factors. The *COL6A1*, *COL6A2*, and *COL6A3* genes encode the three primary alpha chains alpha 1, alpha 2, and alpha 3 that assemble to form collagen type VI [1].

Pathogenic variants involving any of these three genes disrupt the assembly or function of collagen VI, leading to a phenotypic continuum of neuromuscular disorders known as collagen VI‐related dystrophies (COL6‐RDs). The clinical severity of these disorders hinges on the extent of extracellular matrix disruption. At the milder end of the spectrum is Bethlem myopathy, historically described as an exclusively autosomal dominant condition, while the more severe end is represented by Ullrich congenital muscular dystrophy (UCMD), which can result from either recessively or dominantly acting mutations [2, 3].

First described by Otto Ullrich in 1930, UCMD is a rare, severe genetic disorder with an estimated prevalence of 0.13 per 100,000 [4]. It is clinically characterized by a paradoxical presentation: early onset generalized hypotonia and severe proximal joint contractures, accompanied by marked distal joint hyperlaxity. Most patients experience significant motor delays, and disease progression inevitably leads to restrictive respiratory failure, which remains a primary cause of morbidity and mortality in the first two decades of life [5]. Currently, there is no curative treatment for UCMD. Management relies heavily on a proactive multidisciplinary approach, focusing on supportive care, early respiratory monitoring, and intensive physical therapy to manage contractures and preserve motor function.

Despite advancements in genomic diagnostics, the identification of novel missense mutations frequently results in the classification of Variants of Uncertain Significance (VUS), complicating definitive diagnoses [6]. Documenting these variants alongside comprehensive clinical phenotyping is essential for eventual variant reclassification, especially in underrepresented patient populations. Herein, we report a rare case of a novel *COL6A1* variant (c.2821C>T; p. Leu941Phe) presenting classic UCMD features in a child with congenital hypotonia and global developmental delay. This report was prepared following the CARE guidelines for medical case reports [7].

## 2 Case History/Examination

A 3‐year‐old male patient, born at term following an uncomplicated gestation via normal spontaneous vaginal delivery (NSVD). His birth parameters were within normal limits, including a weight of 3.300 kg, length 51 cm, and occipitofrontal circumference 36.5 cm. He was born to consanguineous first‐cousin parents.

Abnormal neuromuscular features were noted early in infancy. At approximately 1 month of age, during hospitalization for an acute illness, a physical examination revealed dysmorphic features such as low‐set ears, a high‐arched palate, and a low anterior hairline. Musculoskeletal abnormalities were also noted, consisting of clenched fingers more noticeable on the right side and a congenital clubfoot deformity (without documented laterality) (see Figure 1). Longitudinal developmental follow‐up was prompted by these findings.

**FIGURE 1:**
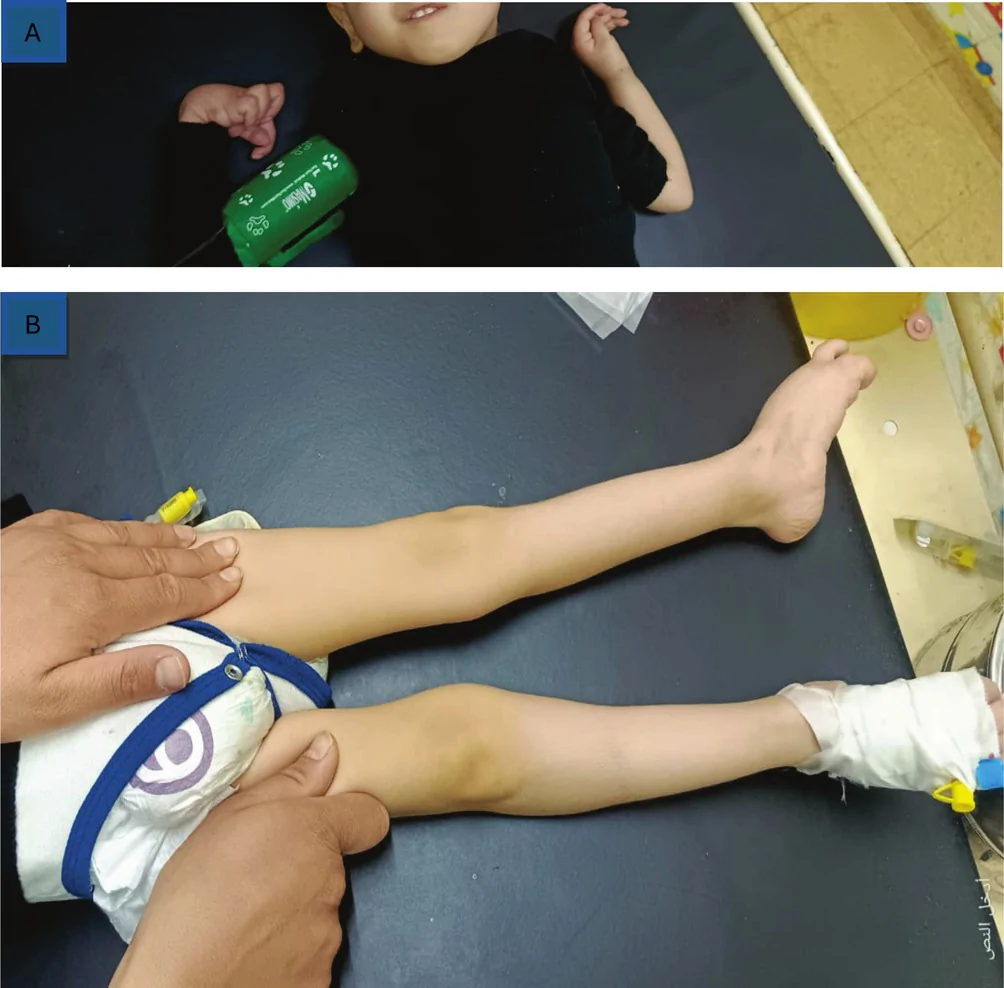
Clinical features of the patient with Ullrich congenital muscular dystrophy. The images demonstrate generalized hypotonia with a characteristic posture, proximal muscle weakness, and distal joint hyperlaxity. Lower limb examination shows reduced muscle bulk and hypotonia with increased joint mobility at the ankles. Facial features are anonymized to preserve patient confidentiality.

## 3 Differential Diagnosis, Investigations and Treatment

Developmental assessment revealed a significant global delay. Gross motor development was markedly affected; at his current age, the patient is unable to sit independently, although he can sit with support. There is no history of developmental regression. While his fine motor function is asymmetric, he has difficulty using his right hand to reach for objects. The left hand is relatively better preserved, though also partially affected. He is still nonverbal, so language development is severely delayed; however, receptive language seems to have been spared, as evidenced by his consistent response to simple commands and his name. With appropriate eye contact and social smiles, social development is largely maintained. On neurological examination, generalized hypotonia was noted; both axial and appendicular involvement were noticed. More distal muscle weakness was observed than proximal muscle weakness. There were fewer voluntary movements and clenched fingers in the upper limbs, which were particularly noticeable on the right side. Deep tendon reflexes were found to be globally absent. There were no clinical signs of lower motor neuron involvement, such as tongue atrophy or fasciculation. There were no reports of seizures and no issues with hearing or vision. At the time of review, current growth parameters were unavailable. Considering the global developmental delay, hypotonia, and progressive joint contracts exhibited by the patient and consanguineous parents, there was suspicion for a genetic cause early in the diagnostic workup. Therefore, whole exome sequencing was undertaken as a diagnostic test. A homozygous variation of unknown significance (VUS) in the COL6A1 gene (c.2821C>T; p. Leu941Phe), which is linked to congenital muscular dystrophies related to collagen VI, was discovered by WES. This variant has been submitted to the ClinVar database and has been assigned accession number SCV007518538. Segregation analysis was not performed due to the unavailability of parental genetic testing. Serum creatine phosphokinase (CPK) levels were measured as part of the neuromuscular evaluation and found to be within the normal reference range (78 and 114 U/L; reference range 0–190 U/L). Based on genetic identification, and to better delineate the neuromuscular phenotype, the patient also proceeded with neurophysiological studies, such as nerve conduction studies (NCS) and electromyography (EMG). NCS showed decreased compound motor action potential amplitudes in several motor nerves. The response of the left median motor nerve was absent, and the right median motor nerve showed reduced amplitude with decreased conduction velocity. Median nerve studies interpretation was limited by hand deformities and joint contractures. EMG showed normal spontaneous activity, whereas early recruitment of small motor units was noted in the tested left tibialis anterior muscle. The general neurophysiological impression was represented by non‐specific myopathic changes. The assessment of global developmental delay also included brain magnetic resonance imaging (MRI), which showed prominence of the anterior horns of the lateral ventricles, widening of the Sylvian fissures, and diffuse reduction in white matter volume. These results were described as non‐specific.

## 4 Discussion

Collagen VI‐related dystrophies are notoriously heterogeneous. The classical presentation of UCMD involves early‐onset hypotonia, delayed motor milestones, proximal joint contractures, and striking distal hyperlaxity [4]. In our patient, the paradoxical combination of significant distal hypermobility and progressive proximal contractures, combined with an absence of deep tendon reflexes and a normal CPK level, strongly points toward a clinical diagnosis within the UCMD spectrum. While the global prevalence of UCMD is estimated at 0.13 per 100,000 [4], specific epidemiological data regarding COL6‐RDs in the Middle East remain remarkably scarce. The Arab population, including Palestine, is characterized by high rates of consanguineous marriages, which significantly increases the incidence of rare autosomal recessive conditions. In our patient, the consanguineous background (first‐cousin parents) directly contributed to the expression of this rare homozygous mutation. Documenting such cases in the Middle East is therefore crucial; it not only expands the global mutational spectrum of *COL6A1* but also highlights the need for targeted genetic counseling and screening in highly consanguineous communities.

Genetically, the patient harbors a homozygous missense variant in *COL6A1* (c.2821C>T, p. Leu941Phe). While current ACMG guidelines classify this specifically as a VUS due to a lack of previously published functional and population data [6], pathogenic missense variants affecting highly conserved residues in the triple‐helical domains of collagen are well‐established mechanisms of UCMD [8]. For instance, similar novel homozygous *COL6A1* missense variants, such as p. Gly556Val, have been previously identified as the direct cause of classic UCMD features in consanguineous families [8]. Furthermore, mutations in non‐coding regions and splice‐site variants in *COL6A1* have also been proven to disrupt collagen VI formation, even when initially designated as VUS [9]. It is noteworthy that our patient presented with a significant global developmental delay, particularly in expressive language, which is generally atypical for COL6‐RDs as cognitive function is classically spared. The whole exome sequencing (WES) did not identify any additional secondary genetic variants to explain this delay. Therefore, these atypical features might be attributed to the diffuse reduction in white matter volume observed on the brain MRI, or potentially secondary to the early severe motor impairment and limited environmental interaction, though an independent co‐occurring etiology cannot be entirely ruled out.

The differential diagnosis for a floppy infant with delayed motor milestones and joint contractures is broad. A primary consideration is Spinal Muscular Atrophy (SMA). However, the patient's lack of tongue fasciculations, intact receptive language, and the specific EMG finding of myopathic (rather than neurogenic) changes make SMA highly unlikely [10]. Other congenital myopathies and muscular dystrophies were also considered. Yet, the normal CPK levels effectively rule out most dystrophinopathies and alpha‐dystroglycanopathies, which typically present with markedly elevated CPK [11]. The distinct structural joint abnormalities clenched fingers and clubfoot further align the phenotype specifically with collagen VI deficiencies rather than other central or peripheral causes of hypotonia.

Given the lack of definitive targeted therapies, the clinical management of this patient focuses on a supportive, multidisciplinary strategy. Current interventions include regular physical and occupational therapy aimed at minimizing the progression of proximal contractures and optimizing his limited motor abilities. Furthermore, considering the natural history of UCMD, routine pulmonary function monitoring and proactive surveillance for nocturnal hypoventilation have been established as essential components of his long‐term care plan. This case presentation acknowledges certain limitations. Primarily, the absence of familial segregation testing and functional studies specifically muscle biopsy, or collagen VI immunohistochemistry on a skin biopsy or fibroblast culture limits our ability to definitively upgrade the variant from VUS to pathogenic according to strict ACMG criteria [6]. Additionally, muscle imaging (such as whole‐body or lower limb muscle MRI), which can reveal characteristic patterns of fibro‐fatty replacement in COL6‐RDs, was not performed. This was largely due to resource constraints and the significant clinical risks associated with administering general anesthesia or deep sedation to a pediatric patient with severe hypotonia for a prolonged scan.

Nevertheless, the strong genotype–phenotype correlation observed here remains highly valuable. Documenting novel variants in populations with high rates of consanguinity is a critical step in expanding global genomic databases. Clinical reports such as this provide the necessary phenotypic evidence that facilitates the future reclassification of VUS, ultimately improving diagnostic certainty and genetic counseling for affected families.

## 5 Conclusion and Results (Outcome and Follow‐Up)

This report describes a child presenting with the classic clinical hallmarks of Ullrich congenital muscular dystrophy, harboring a novel homozygous missense variant in the *COL6A1* gene (c.2821C>T; p. Leu941Phe). While currently classified as a VUS, the compelling phenotypic overlap characterized by congenital hypotonia, joint contractures, and normal CPK levels strongly supports its pathogenicity. This case underscores the necessity of detailed clinical phenotyping to aid in the continuous refinement and reclassification of novel genomic variants, particularly in underrepresented populations.

## Author Contributions

**Khadeeja Nofal:** investigation, data curation, writing – original draft. **Hamza M. A. Ghaith:** investigation, data curation, writing – review and editing. **Haneen Abdullatif Barghouthi:** investigation, writing – review and editing, data curation. **Mohammad Shahrori:** formal analysis, validation, writing – original draft. **Batool Darraj:** conceptualization, methodology, writing – review and editing. **Anwar Abu Hetta:** conceptualization, investigation, data curation, supervision.

## Funding

The authors have nothing to report.

## Consent

Written informed consent for publication was obtained from the patient's legal guardians.

## Conflicts of Interest

The authors declare no conflicts of interest.

## Data Availability

All relevant data are included within this article. The identified COL6A1 variant (NM_001848.3: c.2821C>T; p. Leu941Phe) has been submitted to the ClinVar database and is available under accession number SCV007518538.
